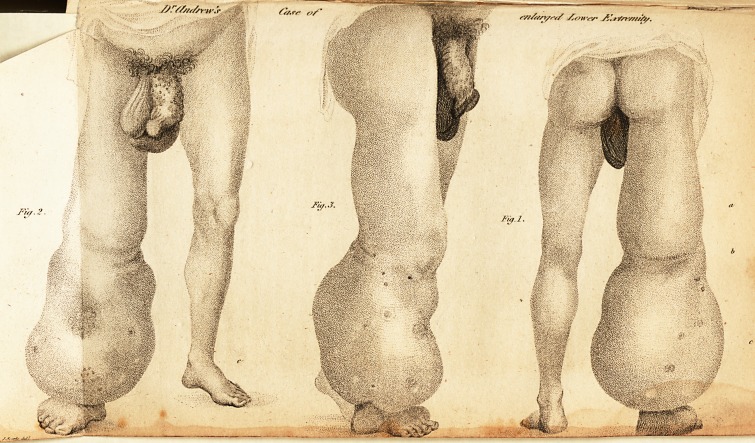# Dr. Andrews on an Enlarged Lower Extremity

**Published:** 1811-05

**Authors:** M. Andrews

**Affiliations:** Madeira


### *To the Editors of the Medical and Physical Journal.*

(With an Engraving.)

Gentlemen,

I HAVE taken this opportunity of communicating the following case to you, and shall be glad to have your remarks on it, and also those of such other practitioners as may have met with similar cases.

The disease comes near to that which is called the Barbadoes leg, but differs from it in some essential points.

As the history of the case alone would be imperfect, I have accompanied it with drawings, which I hope will furnish every satisfactory particular relative to it.

Gentlemen,

Yours,

M. ANDREWS, M.D.*

*Madeira, June* 1, 1810.

---

Manoel Francisco, a native of the Western Islands, 24 years of age, gave me the following history of his complaint: He says, he is informed by his parents, that from his infancy he has had a thickening of the prepuce, and a peculiar enlargement of the scrotum which ulcerated and discharged a thin ichorous fluid; he had also a fistulous opening immediately behind the glans penis through the prepuce, by which a considerable proportion of urine passed every time he made water, but without pain.

From his infancy also, he has been subject to attacks of erisypelatous inflammation, confined to the right limb; they were so far to be considered periodical, as coming on generally when the *moon was at the full*, but not regularly at *those periods*.

These attacks commenced at the right hip, and extended down the leg to the toes; at those periods he experienced pain in the limb, particularly at the knee joint, accompanied a slight degree of fever but no swelling: they seldom lasted longer than three or four days.

About six years since, the scrotum inflamed, and suppurated,

---

* The Editors are obliged to Dr. Adams for the communication of this history of an uncommon enlargement of the lower extremity. As Dr. Adams is particularly versant in this disease, they hope to have a further elucidation of it from his pen.

Dr. Andrew's Case of enlarged Lower Extremity. [illegible]

rated, the abscess opened in three places, and discharged a considerable quantity of pus ; these openings were healed by local applications in about four months ; but the thickening of the scrotum and prepuce encreased, and the latter became covered with small warty excrescences, from which occasionally oozed a thin transparent fluid.

From the time the openings in the scrotum were healed, the swelling of the limb first took place, beginning at the foot, and extending itself upwards to the hip ; from which period he has been free from pain in the limb.

The great increase of size has been within the last year ; several small points of ulceration began in different parts of the leg, but soon terminated in a dry scurf, not however of a furfuraceous kind.

The man's health is exceeding good, his features regular and rather handsome than otherwise, has the usual quantity of hair on the pubes, is able to bend the knee or ankle joints with perfect ease, and, excepting the additional exertion necessary to draw so extraordinary a weight, walks apparently with little inconvenience. He has for several years been at sea, and is able to go to the mast head, and discharge his duty, with as much alacrity as any other seaman.

He assured me that his parents have always been very healthy.

The spermatic chord on the left side appears to be perfect, and the testicle, though smaller than usually met with in adults, is to be distinctly felt ; on the right side, the chord only is distinguishable, but not the testicle, and appears to the feel, more like a loose skein of cotton between the finger and thumb.

On questioning him relative to his desires for the female sex, he assured me that he never has felt any, nor does it appear probable that he could complete the act, as, from the thickened and elongated state of the prepuce, the glans penis cannot be discovered. The circumstances worthy of remark in this case are, that the skin of the leg does not give the appearance of disease ; if you except the few spots that ulcerated, every other part appears like a distended healthy skin, and has none of the characteristic marks of the elephantiasis or Barbadoes leg. There is no enlargement of the inguinal glands or any appearance of tumour in the thigh, and from the time the limb began to encrease in size, he has been perfectly free from pain in it.

## Figures and Tables

**Fig. 2. Fig.3. Fig.1. f1:**